# Coinfection of *Strongyloides stercoralis* and *Aspergillus* sp.

**DOI:** 10.1155/2020/8649409

**Published:** 2020-05-26

**Authors:** Marjan Motamedi, Lida Haghighi, Mostafa Omidian, Bahador Sarkari

**Affiliations:** ^1^Department of Parasitology and Mycology, School of Medicine, Shiraz University of Medical Sciences, Shiraz, Iran; ^2^Basic Sciences in Infectious Diseases Research Center, Shiraz University of Medical Science, Shiraz, Iran

## Abstract

**Background:**

*Strongyloides stercoralis* has the ability to proliferate in its hosts for a long time. In most patients with a competent immune system, the infection remains asymptomatic.

**Objectives:**

Herein, we report a case of concomitant infection of *Strongyloides* and *Aspergillus*. Similar cases reported previously were reviewed in the literature and discussed in terms of diagnosis, clinical presentation, and treatment.

**Methods:**

The patient was a 55-year-old man who had a medical history of two masses in his lung and was treated with corticosteroids six months before the presentation.

**Results:**

Using the parasitological methods, massive actively motile larvae of *S. stercoralis* were seen in the patient's faecal sample. *Aspergillus* infection was isolated from his fresh bronchoalveolar lavage (BAL) sample and confirmed by observing the septate, dichotomously branched hyphae in direct microscopic examination and also the isolation of the fungus from the culture medium. Molecular analysis revealed that the fungal species isolated from the patient are *A. flavus* and *A. niger. Conclusion*. The case highlights the features of concomitant infection of *S. stercoralis* and *Aspergillus* in immunocompromised patients and the importance of screening patients for strongyloidiasis before initiation of immunosuppressive therapy.

## 1. Background

Strongyloidiasis is a parasitic infection, mainly caused by *Strongyloides stercoralis* [1]. *S. stercoralis* is a globally distributed nematode that causes clinical symptoms in humans. It is estimated that around 370 million people, mainly in tropical and subtropical regions of the world, are infected with this nematode [2], and it is believed that the number of cases is increasing worldwide [3, 4].

The parasite has a complicated life cycle which includes a free-living cycle in soil and a parasitic phase in the host. Parasite transmission to humans occurs mainly through the entry of filariform larvae into the body via skin contact, although transmission may also occur through unusual ways, such as organ transplantation [5, 6]. After entering the body, the filariform larvae enter the lungs through the bloodstream and enter the gut through swallowing and become the parthenogenetic female.

*S. stercoralis *can survive for many years in the body without any clinical symptoms. However, disseminated disease or hyperinfection syndrome may develop as a result of immunocompromised states like administration of corticosteroids, tumor necrosis factor-antagonists, and transplantation, which carries a mortality rate of up to 87% [7]. Disseminated disease or hyperinfection syndrome may also occur in individuals coinfected with HTLV and alcoholics. Here, we describe the coinfection of *S. stercoralis* and *Aspergillus* in a patient who was treated with steroids for having two masses in his lung. Moreover, similar cases reported in the literature have been searched for and addressed in this report.

## 2. Case Report

A 55-year-old man originally from Behbahan (a county in Khuzestan Province in the south of Iran) was admitted to the Department of Internal Medicine, in Martyr Faghihi Hospital, Shiraz, Iran, in June 2019. He presented with chronic cough, shortness of breath, wheezing, chest pain, obvious weight loss, weakness, and loss of appetite but without chills or night sweats. He was a worker at the cement factory and had a history of picking mushrooms in the forest with bare feet for the last 8 months before the current admission. His previous medical record was indicating that he had two masses in his right lung for the past one year. Pantoprazole, beclomethasone, and atrovent therapy had been initiated for his chronic pneumonia before the current presentation. Moreover, methylprednisolone (600 mg/8 h; IV) and beclomethasone (two puffs, two times a day; each puff contains 40 *μ*g of beclomethasone) were given to the patient before and during hospital stay.

A computed tomographic scan of his chest revealed two masses along with moderate to severe pleural effusion and evidence of pneumothorax.

Initial laboratory indices were as follows: hemoglobin concentration of 10.1 g/dL, white blood cells counts of 33.88 (normal, 4.0–10.0) *×*  10^9^ cells/L with differentials of 31.81% neutrophil, 0.6% lymphocytes, 1.4% monocytes, zero count of eosinophil, and platelets count of 813 (normal, 150–450) *×* 10^9^ cells/L. He had an increased C-reactive protein (CRP) level of 118 (normal, <10) mg/L and erythrocyte sedimentation rate (ESR) of 114 (normal 0–20) mm/hour. His creatinine concentration (3.7 mg/dL) was above the normal range (0.7–1.4 mg/dL).

**Figure 1:**
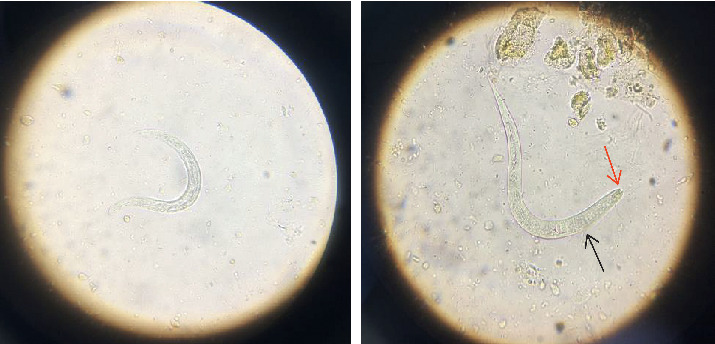
Larva of *Strongyloides stercoralis* in an unstained wet mount of stool, isolated from the patient in the current study (400x), where red arrow indicates short buccal canal and black arrow indicates rhabditoid esophagus.

Routine parasitological laboratory analysis of the fresh faecal specimen was requested, and massive (up to 10 larvae per microscopic field; 10X) actively motile larvae of *S. stercoralis* were identified in the wet mount preparation. These larvae are about 0.20–0.35 mm long with a round tip and elongated tail. They have a short buccal canal, an esophageal bulb, and a prominent genital primordium (Figure 1).

**Figure 2:**
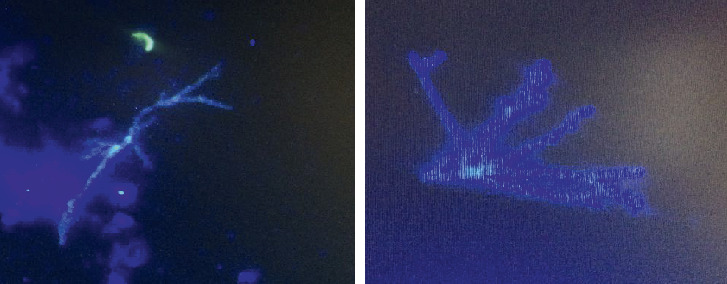
Fluorescent images of Calcofluor white-stained dichotomous branching hyphae in the BAL sample of the patient in the current study.

**Figure 3:**
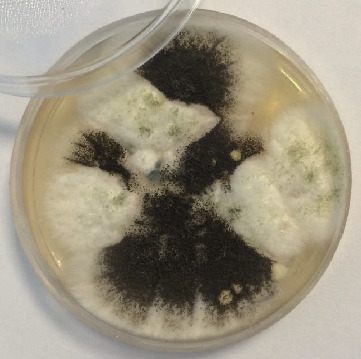
Culture of the BAL revealed the presence of *Aspergillus* species colonies (*A. flavus and A*. *niger*).

Subsequently, bronchoalveolar lavage (BAL) and urine samples were subjected to direct microscopic examination in terms of *S. stercoralis* infection, but the samples were not positive for *S. stercoralis*. Surprisingly, a direct examination of BAL revealed septate hyphae with regular dichotomous branching. A potassium hydroxide-Calcofluor mount of BAL confirmed the presence of septate, dichotomously branched hyphae (Calcofluor white bind to chitin that is present in the cell walls of fungi and fluoresces when exposed to UV light) (Figure 2). The patient's BAL sample was cultured in a plate containing sabouraud dextrose agar, with 0.05% chloramphenicol. Two identical species of *Aspergillus* grew abundantly on the plate at the sample inoculation points (Figure 3).

**Figure 4:**
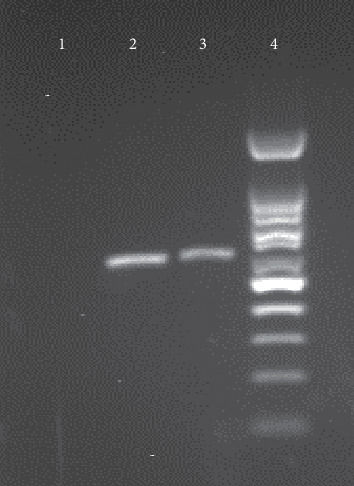
Agarose gel electrophoresis of ITS PCR products. Lane 1, negative control, lane 2, *Aspergillus flavus*, lane 3, *Aspergillus niger*, and lane 4, a 100 bp molecular size marker.

The fungal colonies were isolated from the plate, and molecular tests were performed for accurate identification of *Aspergillus* species, as previously described [8]. In brief, DNA was extracted from the isolates, using homogenization by a rapid mini-preparation method [9]. Polymerase chain reaction (PCR) amplification of the ITS1–5.8S–ITS2 rRNA region was performed, using a panfungal primer pair including ITS1 (5′-TCCGTAGGTGAACCTGCGG-3′) and ITS4 (5′-TCCTCCGCTTATTGATATGC-3′). The PCR amplified an approximately 550–600 bp fragment in both of the tested *Aspergillus* species.  Figure 4 shows the agarose gel electrophoresis of PCR products from the isolated *Aspergillus* species.

Sequencing was performed, using the forward primer (ITS1) as for the PCR, via an automated DNA sequencer (ABI PrismTM 3500 Genetic Analyzer, Genetic Group). The sequences were edited with Geneious software (http://www.geneious.com), and for final identification, the obtained consensus sequences were compared with those of available sequences in the GenBank (https://www.ncbi.nlm.nih.gov/pubmed/). The comparative DNA sequence analysis showed that the amplified sequence of one of the colonies had 99% identity with the ITS regions of *A. niger* with GenBank accession number MH055397.1 and another colony sequence had 100% identity with the sequence of *A. flavus* (GenBank accession number: MN179296.1). The consensus nucleotide sequence data determined in this study were deposited in the GenBank, under the accession numbers of MT197480 and MT197481.

Cytological evaluation of the pleural fluid sample also did not reveal any sign of malignancy. The patient continued to be in severe hypoxic and respiratory failure without improvement and eventually developed cardiac arrest and passed away 9 days after admission. Anthelmintic therapy (ivermectin; 200 *µ*g/kg per day, orally) was started for the patient for one day, but due to his severe condition, the treatment was not continued. Postmortem examination was not allowed.

## 3. Discussion and Review of the Literature

Strongyloidiasis usually causes a symptom-free chronic disease that can remain undetected for a long time. However, in immunosuppression status, it may cause fatal hyperinfection syndrome or disseminated infection [10]. Accordingly, it has been recommended that in the endemic areas, patients undergoing immunosuppressive drug therapy need to be serologically tested for *Strongyloides* infection before starting treatment [11, 12], although serological diagnosis has its own limitations and is not widely available in every treatment center.

The most common concomitant infection in disseminated strongyloidiasis is Gram-negative bacteria that are carried by the parasite larvae from the gut to the bloodstream or lung [13, 14]. Coinfection of *S. stercoralis* and fungi, especially with *Aspergillus,* is rarely reported [15, 16]. *Aspergillus* is a worldwide distributed fungus that causes disease in immunocompromised patients and patients with underlying lung disease.

We performed a literature search using the terms “*Strongyloides stercoralis* AND *Aspergillus*,” “Strongyloidiasis AND Aspergillosis,” on PubMed, Scopus, and Google Scholar. Inclusion criteria were the diagnosis of *S. stercoralis* infection, disseminated or hyperinfection syndrome, confirmed by detection of larvae, and detection of *Aspergillus* by conventional laboratory methods (direct microscopic examination or culture). Exclusion criteria were lack of a parasitological or mycological confirmation. The study variables were age, gender, place of origin, underlying diseases, immunosuppressive therapy, clinical manifestations, eosinophilia, diagnostic evidence for *S. stercoralis* and *Aspergillus*, determination of *Aspergillus* species causing the infection, antiparasitic treatment, and the main outcomes. In total, 6 studies met the inclusion criteria, describing the coinfection of *S. stercoralis* and *Aspergillus* [15–20]. The clinical and laboratory details of each patient are given in Table 1.

Of these 6 reported cases, the majority (*n* = 5; 83.3%) were male and the median age was 61 years (ranged from 36 to 74). The age of our patient in the current study was in line with the age of the previously reported cases [16, 19].

Four (66.6%) of the reported cases, as well as our case, were admitted with a history of pulmonary abnormalities. Other underlying diseases that have been observed include acute lymphoblastic leukemia and ulcerative colitis [17, 18].

The use of corticosteroids is an important risk factor of strongyloidiasis. Prolonged corticosteroid therapy was the attributable cause of severe strongyloidiasis in 83.3% of the reported cases. Cases 2 and 5 (Table 1) had also received chemotherapy and radiotherapy, respectively, besides the use of the corticosteroid [17, 20]. Our patient had been given corticosteroids (methylprednisolone and beclomethasone) for 1 year due to his pulmonary mass. The administration of corticosteroids has long been a mainstay of therapy for inhibition of tumor growth [21]. Considering the comorbidity of strongyloidiasis with other conditions, a recent systematic review described a large number of severe strongyloidiasis cases (67%) as receiving steroids, having HIV infection (15%), or being transplant recipients (11.5%) before the onset of symptoms [22].

Clinical manifestations in our case included fever, a poor appetite, and weight loss, which is consistent with the findings of other reported cases [15–17, 19]. Only two (33.3%) cases presented with digestive disorders which is one of the main symptoms of hyperinfection in strongyloidiasis [18, 20].

Eosinophilia is usually defined as an eosinophil count of more than 5% of the circulating leukocytes [23]. Boulware et al. reported that almost 84% of strongyloidiasis cases have an eosinophil rate of >5% [24]. However, the immunocompromised patient with heavy *S. stercoralis* infection may have a normal or even a reduced eosinophil count [16]. Thus, a parasitic infection with normal or less than normal eosinophil levels is not unexpected. Our case had no eosinophilia which was similar to findings in cases 4 and 5 [15, 20].

In most of the reported cases, the initial diagnosis has not been strongyloidiasis, but after the evaluation of duodenal biopsy, bronchoalveolar lavage (BAL), and faecal or sputum samples, *S. stercoralis* infection has been confirmed [15–18, 20, 25]. In our case, we first did not think of a parasitic infection until we took the result of wet mount preparation of the fresh faecal sample.

In our case, *S. stercoralis* diagnosis was made by the unstained wet mount of the patient's stool sample. The most sensitive method for parasitological diagnosis of *S. stercoralis* is the agar plate culture, yet this method is mostly available in the research centers [26]. The direct wet mount microscopic examination is not the most adequate method for *S. stercoralis* diagnosis, since it has a low sensitivity. The fact that the larva was detected by this method in our case, by itself, already indicates that there was a high parasitic load.

*Aspergillus* species involvement in disease pathology must be carefully considered since these fungi have a universal distribution and are common laboratory contaminants. BAL, bronchial wash (BW), and sputum are the frequently used samples that are subjected to direct examination and culture for the confirmation of fungal infection. The criteria for diagnosis are important for distinguishing contaminants and pathogenic molds. The majority of studies stated at least three criteria which included identification of the mold in the sample by microscopy, isolation in culture, repeated isolation in culture, and inoculums counting [27]. Diagnostic evidence for *Aspergillus* infection in our case was the identification of septate, dichotomously branched hyphae in direct microscopic examination of fresh BAL sample and isolation of the fungus on chloramphenicol containing medium in at least 5 inocula. Studies have shown that isolation of *Aspergillus* from sputum in immunocompromised patients has a positive predictive value of 80–90% for the presence of invasive pulmonary aspergillosis (IPA) [28, 29]. IPA is caused by one of the four species of *Aspergillus*: *A. fumigatus*, *A. flavus*, *A. niger*, and *A. terreus*. We have isolated both *A. flavus* and *A. niger* species from the BAL sample in our case while as listed in Table 1, in three cases, *A. fumigatus* caused the infection [16, 19, 20]. This discrepancy may be due to the fact that in Iran, *A. flavus* is the dominant species of *Aspergillus* which has been isolated from clinical samples because of the abundance of the spores in the environment of this species compared to other species [30]. Although we have isolated both *A. flavus* and *A. niger* species from the patient's sample, we believe that the *A. flavus* is the main species of *Aspergillus* that caused pathogenesis in the patient and the possibility of environmental contamination by *Aspergillus niger* cannot be simply ruled out.

It should be noted that in our case, as the patient already had two lung masses and had a history of lung disease, the possibility of the infection with *Aspergillus* sp. occurring before the *S. stercoralis* infection cannot be ruled out.

Among the six reported cases in the medical literature, 3 (50%) cases have been discharged in stable condition, 1 (16.6%) case had fatal outcomes because of cardiac arrest, and 2 (33.3%) cases passed away without reporting the cause. It should be noted that among the six reported cases (Table 1) who passed away (*n* = 3, 42.8%), there were two cases who had not received any or proper antiparasitic treatment and one case who had taken a drug other than ivermectin (thiabendazole).

## 4. Conclusion

In this report, we have shown evidence that the use of corticosteroids may result in an immunosuppression status that makes the patients prone not only to common pathogen agents but also to more unusual pathogens, for instance, *S. stercoralis* and *Aspergillus*. It can be recommended that, in the endemic areas, before initiating any immunosuppressive therapy, routine faecal screening needs to be performed. Moreover, prophylactic therapy, when diagnostic techniques are unavailable, might be considered.

## Figures and Tables

**Table 1:** Reported cases of coinfection of *Strongyloides stercoralis* and *Aspergillus*.

	Age/sex	Place of origin	Underlying disease	Immunosuppressive therapy	Clinical presentation	Eosinophilia	Positive sample for *S. stercoralis*	Positive sample for *Aspergillus*	*Aspergillus* species identified	Antiparasitic treatment	Outcome	Reference
1	55 year/man	Behbahan	Two mass in the lung	Oral corticosteroid	Chronic cough, shortness of breath, wheezing, chest pain, obvious weight loss, weakness, and loss of appetite	0%	Wet mount of faeces	Sputum direct examination and culture	*A. flavus* and *A. niger*	Ivermectin (only for one day)	Developed cardiac arrest and deceased	This study
2	36 year/man	San Antonio, Texas	Acute lymphoblastic leukemia	Oral corticosteroid and chemotherapy	Dyspnea, malaise, fever, and hemoptysis	18% eosinophilia	Sputum cytology	Sputum and lavage fluid cultures	*A. flavus* and *A. terreus*	Ivermectin	Discharged in stable condition	Shrestha et al. [17]
3	66 year/man	Nicaragua	Ulcerative colitis	IV steroids	Watery diarrhea with occasional blood	Unknown	BAL and CSF direct examination	BAL direct examination and at autopsy, multiple foci of lung involvement by *Aspergillus* were seen	Unknown	Ivermectin	Developed bradycardia and asystole and deceased	Imperatore et al. [18]
4	74 year/man	Unknown	Cough, shortness of breath after activity expectoration	Unknown	Weight loss, hemoptysis accompanied by fever, and poor appetite	0.1% eosinophilia	BAL direct examination and wet mount of faeces	Several sputa and BAL cultures	Unknown	Albendazole	Discharged in stable condition	Guo et al. [15]
5	73 year/man	Unknown	Non-Hodgkin's lymphoma, ischaemic heart disease, and chronic obstructive pulmonary disease	Oral corticosteroid and radiotherapy	General malaise, high fever, vomiting, and low blood pressure	1% eosinophilia	Sputum direct examination	Sputum direct examination and culture	*A. fumigatus*	Did not receive	Deceased	Wagenvoort et al. [20]
6	58-year/woman	Unknown	Nonallergic asthma	Aerosol corticosteroids	Progressive respiratory distress syndrome	17.2% eosinophilia	Tracheal aspirations	High serum level of antibodies to *Aspergillus*	*A. fumigatus*	Ivermectin	Discharged in stable condition	Jacquemart et al. [19]
7	59-year/man	Unknown	Chronic obstructive lung disease	Oral corticosteroid	Hematuria, hemoptysis, hematemesis, and respiratory failure	Unknown	Wet mount of faeces and sputum direct examination	Sputum culture and at autopsy demonstrated disseminated aspergillosis	*A. fumigatus*	Thiabendazole	Deceased	Tankanow et al. [16]

## Data Availability

The data used to support the findings of this study are included within the article.
